# Tumoral and non-tumoral trachea stenoses: evaluation with three-dimensional CT and virtual bronchoscopy

**DOI:** 10.1186/1749-8090-2-18

**Published:** 2007-04-12

**Authors:** Efstratios N Koletsis, Christine Kalogeropoulou, Eleni Prodromaki, George C Kagadis, Konstantinos Katsanos, Konstantinos Spiropoulos, Theodore Petsas, George C Nikiforidis, Dimitris Dougenis

**Affiliations:** 1 Department of Cardiothoracic Surgery, School of Medicine, University of Patras, Greece; 2 Department of Radiology, School of Medicine, University of Patras, Greece; 3 Department of Pneumonology, School of Medicine, University of Patras, Greece; 4 Department of Medical Physics, School of Medicine, University of Patras, Greece

## Abstract

**Background:**

We evaluated the ability of 3D-CT and virtual bronchoscopy to estimate trachea stenosis in comparison to conventional axial CT and fiberoptic bronchoscopy, with a view to assist thoracic surgeons in depicting the anatomical characteristics of tracheal strictures.

**Methods:**

Spiral CT was performed in 16 patients with suspected tracheal stenoses and in 5 normal subjects. Tracheal stenoses due to an endoluminal neoplasm were detected in 13 patients, whilst post-intubation tracheal stricture was observed in the other 3 patients. Multiplanar reformatting (MPR), volume rendering techniques (VRT) and virtual endoscopy (VE) for trachea evaluation were applied and findings were compared to axial CT and fiberoptic bronchoscopy. The accuracy of the procedure in describing the localization and degree of stenosis was tested by two radiologists in a blinded controlled trial.

**Results:**

The imaging modalities tested showed the same stenoses as the ones detected by flexible bronchoscopy and achieved accurate and non-invasive morphological characterization of the strictures, as well as additional information about the extraluminal extent of the disease. No statistically significant difference was observed between the bronchoscopic findings and the results of axial CT estimations (P = 1.0). No statistically significant differences were observed between bronchoscopic findings and the MPR, VRT and VE image evaluations (P = 0.705, 0.414 and 0.414 respectively).

**Conclusion:**

CT and computed generated images may provide a high fidelity, noninvasive and reproducible evaluation of the trachea compared to bronchoscopy. They may play a role in assessment of airway patency distal to high-grade stenoses, and represent a reliable alternative method for patients not amenable to conventional bronchoscopy.

## Background

Bronchoscopy is considered the "gold standard" for the detection and diagnosis of tracheobronchial pathology because it permits direct visualization of the airway lumen. However, bronchoscopy has potentially hazardous complications in the severely ill patients (profound oxygen desaturation in hypoxemic patients, tachycardia, cardiac arrhythmias, endoscopy-induced inflammation of the immunocompromised), and some technical limitations such as inability to evaluate airway calibre and morphology beyond a high-grade stenosis of the bronchial lumen. Furthermore, it is not an examination well-tolerated by all patients [1-4].

On the other hand, spiral CT is a well-tolerated procedure by all patients, and permits rapid data acquisition during a single breath hold. The acquired images provide detailed information regarding the tracheobronchial tree and its pathology. Moreover, two and three-dimensional (2D, 3D) images generated by CT data, provide additional information regarding airway pathology. A variety of computer processing algorithms can be applied in CT acquired data such as: multiplanar reformatting (MPR), shaded surface display (SSD), maximum or minimum intensity projection (MIP), volume rendering techniques (VRT) and more recently virtual endoscopy (VE) [5-12].

Virtual bronchoscopy is the specific application of VE for the tracheobronchial tree. It is not invasive and can produce views similar to those produced by conventional bronchoscopy. It can evaluate the airways beyond a high-grade stenosis and it can be performed in patients who cannnot tolerate bronchoscopy. The aforementioned advantages of the computer-generated images render them as a constant demand for the evaluation of these patients [13-15].

The aim of our study was to evaluate the capacity of the computer generated images (2D/3D images and virtual endoscopy) to accurately depict and characterize tracheal stenoses in comparison with conventional fiberoptic bronchoscopy.

## Methods

Spiral CT was performed in 16 patients who were referred for suspected trachea stenoses. Patient population involved 12 men and 4 women; age range 19–77 years, mean age: 62.3 years. Tumor was the cause of tracheal narrowing in 13 patients, while the rest 3 patients suffered from tracheomalacia secondary to prolonged intubation (Table 1). In addition, 5 normal subjects, who underwent computed tomography of the chest for atypical symptoms and had negative pathology were included as a control group.

**Table 1:** Pathologic diagnoses in 16 patients with stenoses at brochoscopy

**Diagnosis**	**No of patients (n = 16)**	**No of stenosis (n = 21)**
**Malignant diseases (n = 11)**		

Invasion by adjacent cancer		
-Lung cancer	6	6
-Thyroid cancer	2	2
-Esophageal cancer	2	2
Metastases		
-Small cell carcinoma	1	4

**Benign disease (n = 5)**		

Tumors		
-Hamartoma	1	1
-Papillomatosis	1	3
Post tracheal intubation stenoses	3	3

All patients underwent fiberoptic bronchoscopy performed by an experienced pulmonologist, and the findings regarding the presence, localization and numbers of stenoses were used as standards of reference.

Pathologic diagnosis was established by analysis of specimens obtained during bronchoscopy (n = 12) or surgery (n = 1) or by evaluating the patients' clinical history (n = 3).

We applied MPR, VRT and VE techniques in all patients in order to evaluate the tracheobronchial tree and thereafter, we compared the results with the existing transverse CT sections, and bronchoscopic findings.

### Data Acquisition

The CT data sets were acquired with a Somatom Plus 4 Power scanner (Siemens Medical Systems, Erlangen, Germany) at 140 kV and 189 mA, respectively. Spiral CT examinations were carried out at deep inspiration without administration of any contrast medium. In order to minimize the motion artifacts due to inability to breath hold, scanning was performed in the caudo-cranial direction. Prior to the examination the patients were hyperventilated with deep inspirations to improve breath holding.

The scan parameters were 5 mm collimation and 7.5 mm/sec feed, while the total scan time ranged between 20–25 sec. The reconstruction interval was set to 2 mm and the field of view (FOV) was confined to 160 mm.

### Three-dimensional reconstructions and virtual endoscopy

All the spiral CT data sets were transferred to the Medical Imaging unit of the Department of Medical Physics through Gigabit uplink based LAN for image processing which was performed on an Analyze PC 5 workstation (Mayo Foundation).

The volume data were loaded to the workstation and with the application of different filters the objects of interest were subsequently segmented (i.e. trachea, major bronchi, lung parenchyma, tumors etc.). After the segmentation process, the objects of interest were reconstructed using the MPR and VRT algorithms. At last, the VE algorithm was applied in all data sets. The same medical imaging specialist, blinded to the outcome of other examinations, performed all image reconstructions.

The whole procedure, in order to generate the 2-D images (MPR), 3-D images (VRT) and the VE images from the axial CT-slices, required approximately 20–30 min for each patient. Good quality images were generated in all cases.

Two independent radiologists, not informed about the clinical history of the patients and their bronchoscopic findings, evaluated the final volume-rendered images. Finally, the interpretations of the computer rendered images were compared to the axial CT images, and bronchoscopic descriptions.

### Statistical analysis

McNemar Test and Wilcoxon Signed Rank Test were applied to evaluate differences between the two groups. A P value of less than 0.05 was considered statistically significant.

## Results

### Axial CT-slices

A total of 21 tracheal stenoses were depicted during fiberoptic bronchoscopy in all 21 subjects (16 patients with trachea narrowing and 5 normal controls). Both blinded readers observed in the axial CT images the same number of lumen narrowing cases but they did not identify the same stenoses.

In one patient with lung cancer, axial CT didn't demonstrate the presence of the stenosis visualized on bronchoscopy. Both readers couldn't differentiate tumor invasion from the altered anatomy of lower trachea due to previous surgery. On the other hand, in cases with multiple lesions, both readers depicted more lesions than bronchoscopy. There was no statistically significant difference among the bronchoscopic findings and the results of axial CT estimations (P = 1.0). Intraobserver differences were not statistically significant (P = 1.0).

### Multiplanar Reformatting (MPR)

Reader 1 detected twenty-two (22) stenoses, though reader 2 detected twenty-three (23) stenoses compared to the twenty-one (21) visualized on bronchoscopy. This 2D- imaging approach depicted more stenoses in subjects with multiple lesions, while bronchoscopy underestimated them. No statistically significant difference among the bronchoscopic findings and the MPR image evaluation was detected (P = 0.705). Intraobserver differences were not statistically significant (P = 1.0).

### Volume rendering technique (VRT)

Both readers detected a total of twenty-four (24) stenoses compared to the twenty-one proved by bronchoscopy. Using VRT 3D-images, both readers demonstrated a larger number of stenoses in all patients with multiple lesions. No statistically significant difference among the bronchoscopic findings and the VRT image evaluation were detected (P = 0.414). Intraobserver differences were not statistically significant (P = 1.0).

### Virtual endoscopy (VE)

**Figure 1:**
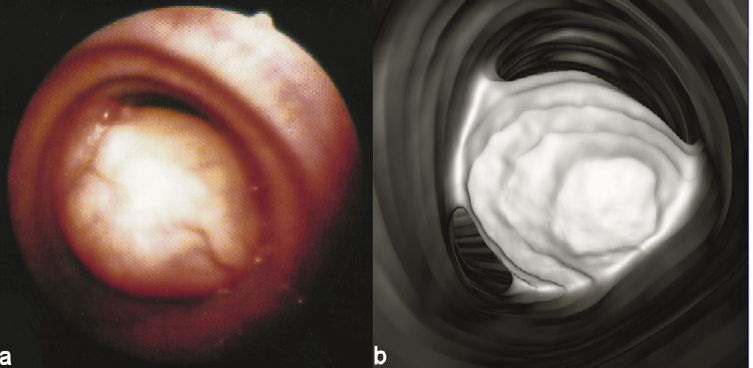
Intraluminal tracheal tumor causing severe airway obstruction. Note the nice correlation between the virtual endoscopic image and the fiberoptic bronchoscopy performed later, which confirmed the diagnosis of a rare case of hamartoma. On one hand fiberoptic bronchoscopy provides the opportunity of biopsy and gross evaluation of the disease, but on the other hand it lacks the ability to navigate distal to an obstructive lesion, like in this case where virtual endoscopy showed a normally patent tracheobronchial tree distal to the hamartoma (images not included).

In our study, virtual endoscopy was very useful in two cases: one patient with papillomatosis, and a second one with a hamartoma (Figure 1) [see Additional file 1], on whom bronchoscopy could not evaluate the lumen of the trachea beyond the narrowed segment, in order to determine the airway patency distal to the lesion. No statistically significant difference among the stenoses depicted by bronchoscopy and the ones observed by VE was detected (P = 0.414). Intraobserver differences were not statistically significant (P = 1.0).

## Discussion

Patients with suspected trancheobronchial pathology typically undergo conventional CT scanning and fiberoptic bronchoscopy (FB). Although both diagnostic modalities (axial CT, FB) are widely accepted for the detection of tracheal stenoses, novel-imaging techniques have emerged to overcome their limitations with comparable sensitivity and specificity values. The objective of our study was to assess the sensitivity of the computer generated images (2D/3D images and virtual endoscopy) in comparison with conventional fiberoptic bronchoscopy.

**Spiral CT**, from the more recent CT scanners, is a well tolerated procedure by all patients and permits rapid data acquisition with high-resolution images of the main lobar and distal segmental airways during a single breath hold. The acquired images provide detailed information regarding the tracheobronchial tree and its pathology. The majority of airway abnormalities are sufficiently evaluated by axial CT images, but there are some limitations of axial images for assessing the airways, such as : (1) limited ability to detect subtle airway stenosis; (2) underestimation of the craniocaudal extent of disease; (3) difficulty displaying the relationships of the airway to adjacent mediastinal structures; (4) inadequate representation of airways oriented obliquely to the axial plane; (5) difficulty assessing the interfaces and surfaces of airways that lie parallel to the axial plane; and (6) generation of a large number of images for review [7,16-18].

The creation of 2-D and 3-D images reformatted from the original axial CT data set can help to overcome these limitations. Such images also augment 3-dimensional perception of the disease, which consequently leads to improved diagnostic confidence of interpretation; enhanced communication among radiologists, clinicians, and patients; and improved pre-procedural planning [6]. By reducing a large axial CT data set into a few images, CT reconstructions also offer clinical physicians the potential advantage of efficiency of review, which is increasingly important in this era of increasing clinical demands in daily practice.

**Figure 2:**
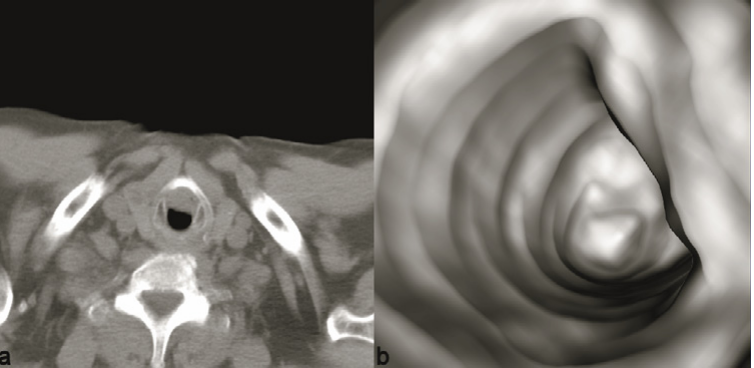
Tracheal stricture after invasion of adjacent thyroid cancer. Axial CT shows parenchymal irregularity and enlargement of the right thyroid lobe. The mass has eroded the right lateral tracheal wall and extended intraluminal to the trachea. Reformatted virtual endoscopy images portray the eccentric tracheal stenosis with an excellent 3-dimensional perceptive.

**Figure 3:**
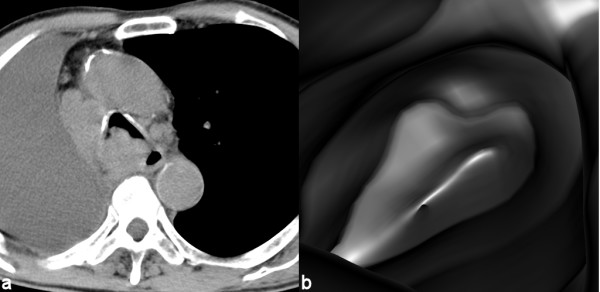
Tracheal invasion of adjacent lung cancer causing severe lumen stricture and distortion. Axial CT provides important information about the extraluminal structures; large fluid collection in the hemithorax, invasion and displacement of the tracheobronchial tree along with mediastinal lymphadenopathy; while reconstructed virtual endoscopic images demonstrate a distorted partially obstructed tracheal lumen. Distal navigation was only available with virtual endoscopy and provided also views of a compromised right main stem bronchus.

Moreover two and three-dimensional (2D, 3D) imaging generated by CT data, provide additional information; both intraluminal and extraluminal; regarding airways anatomy and pathology and has proven to be a well-established method in the diagnosis of those diseases (Figures 2, 3).

A variety of computer processing algorithms can be preformed such as multiplanar reformatting (MPR), shaded surface display (SSD), maximum or minimum intensity projection (MIP), volume rendering techniques (VRT) and virtual endoscopy (VE).

SSD is the most well known technique, where each image voxel is classified as either 0% or 100% (0 or 1) of a tissue type depending on a threshold chosen to display the organ of interest. Its main advantage is that it is a simple process, can be performed on inexpensive computer platforms, and does not require great processing time. Its primary drawback is that the resultant image is related to the selected threshold, thus exploiting only a small part of the volume dataset.

MIP is based on the display of the lowest or highest intensity voxels. Its main disadvantage is the inability to sustain spatial relationships inherent to the data set, while its fundamental advantages are that it requires small processing time and threshold selections are avoided.

In VRT techniques, the entire volume dataset is classified allowing voxels to be clustered into multiple categories on the basis of their attenuation coefficients, and subsequently the clusters are reconstructed as separate anatomic structures. Its primary benefit is that it maintains the original spatial relationships of the volume data. Furthermore, VRT adds depth and enhances details allowing the reproduction of life-like images. However, its main disadvantage is that it requires more computer power and greater processing time than SSD and MIP [4,19-22].

In VE endoluminal surface views produced resemble the ones obtained with fiberoptic bronchoscopy. The main advantage of VE is that it allows perspective volume rendering of the airways without any time-consuming pre-processing. VE is not invasive and can go beyond strictures which the real endoscope cannot overpass (Figues 1, 3).

**Fiberoptic bronchoscopy (FB**) is the best imaging modality for detection and diagnosis of tracheobronchial pathology because it permits direct evaluation of the endoluminal and mucosal lesions of the respiratory tract and can guide biopsies for histologic analysis. However, conventional bronchoscopy has some limitations: (1) it can't pass through severe airway narrowings, (2) it provides scarce information concerning the extent of extraluminal disease and (3) it cannot be tolerated by some patients.

To overcome the limitations of broncoscopy, the abovementioned CT – computed generated images were applied for the evaluation of tracheobronchial pathology.

**Figure 4:**
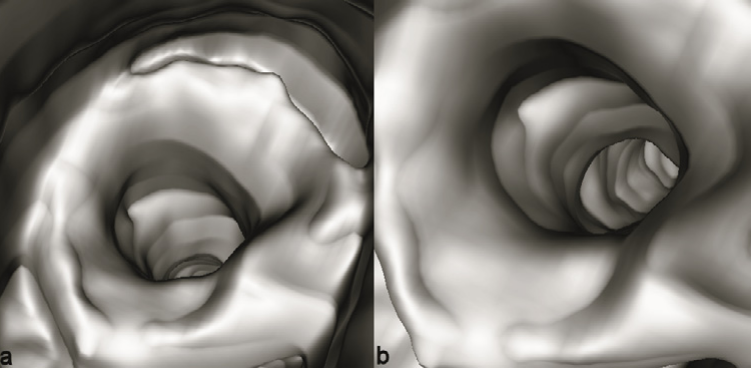
Benign post-intubation tracheal stenosis in a patient with a history of prolonged intensive care unit hospitalization. Virtual bronchoscopy images show a concentric mild lumen stricture with normal patency of the distal airway. In this case invasive fiberoptic bronchoscopy was completely avoided.

**Virtual bronchoscopy **may be used as a complementary imaging technique to conventional bronchoscopy in order to assess the patency of airways beyond a site of high-grade stenoses where the bronchoscope cannot pass [6,23], or in order to provide a navigational aid to improve the yield and safety of transbronchial biopsy [6,24,25]. This sophisticated 3D radiographic imaging technique has even been considered for surveillance of lung malingnancies as a screening program by Wood et al. [26]. It facilitates the evaluation of bronchial lesions from multiple angles overcoming the limits of conventional bronchoscopy [27]. It is an accurate, non-invasive, objective, and readily reproducible imaging modality that may complement conventional diagnostic approaches (axial CT, FB) and may provide a beneficial management of pulmonary patients (Figure 4).

In published literature, CT virtual bronchoscopy is proven to be comparable to FB due to its high specificity, sensitivity and accuracy [28]. According to Xiong et al. [29], the sensitivity of CT based virtual bronchoscopy (ctvb) in detecting central tumors was 93.3% and its accuracy was 93.5%, while according to Hope et al. [30] the methods sensitivity in detecting stenoses of the central airways is 90%, its specificity is 96.6%, and its accuracy is 95.5%.

In our study the computer generated images detected the same stenoses with bronchoscopy, with the advantage that VE found additional stenoses beyond the areas that the bronchoscope could not transverse. These findings can possibly indicate that VE has high diagnostic yield in the setting of multiple lesions. It may provide accurate morphological characterization and mapping of the lesions, helping to plan the most appropriate treatment. Moreover we believe among others that VE may play the role of preliminary study in patients that are not capable to undergo conventional bronchoscopy. In this case the preliminary VE can help to obtain accurate topography of the lesion making the bronchoscopy quicker [5].

## Conclusion

CT and computed generated images may provide a high fidelity, noninvasive and reproducible evaluation of the trachea compared to bronchoscopy. Thoracic surgeons may benefit from the application of three-dimensional and virtual endoscopy imaging, particularly when evaluating patency distal to high-grade stenoses. These techniques may represent a reliable alternative method for patients not amenable to conventional bronchoscopy.

## Abbreviations

MPR: Multiplanar Reformatting

VRT: Volume Rendering Techniques

VE: Virtual Endoscopy

SSD: Shaded Surface Display

MIP: Maximum or minimum Intensity Projection

FB: Fiberoptic Bronchoscopy

## Competing interests

The author(s) declare that they have no competing interests.

## Authors' contributions

All authors: 1) have made substantial contributions to conception and design, or acquisition of data, or analysis and interpretation of data; 2) have been involved in drafting the manuscript or revising it critically for important intellectual content; and 3) have given final approval of the version to be published.

## Supplementary Material

Additional file 1 VE movie of a tracheal tumor causing severe airway obstruction. The video file provided describes the intraluminal tracheal tumor causing the severe airway obstruction of Figure 1. Note the excellent depiction of the main stem bronchi as well as the detailed description of the tracheal tumor both proximally and distally to the lesion. Click here for file
